# Genetic counseling globally: Where are we now?

**DOI:** 10.1002/ajmg.c.31607

**Published:** 2018-03-25

**Authors:** Kelly E. Ormond, Mercy Ygoña Laurino, Kristine Barlow‐Stewart, Tina‐Marié Wessels, Shelley Macaulay, Jehannine Austin, Anna Middleton

**Affiliations:** ^1^ Department of Genetics and Stanford Center for Biomedical Ethics Stanford University Stanford California; ^2^ Department of Pediatrics, College of Medicine University of the Philippines Manila Manila Philippines; ^3^ Cancer Prevention Programs Seattle Cancer Care Alliance Seattle Washington; ^4^ Sydney Medical School Northern University of Sydney Sydney New South Wales Australia; ^5^ Division Human Genetics University of Cape Town Cape Town South Africa; ^6^ Division of Human Genetics, Faculty of Health Sciences University of the Witwatersrand & the National Health Laboratory Service Johannesburg South Africa; ^7^ Departments of Psychiatry and Medical Genetics University of British Columbia Vancouver British Columbia Canada; ^8^ Society and Ethics Research, Connecting Science Wellcome Genome Campus Cambridge United Kingdom; ^9^ Association of Genetic Nurses and Counsellors United Kingdom and Republic of Ireland; ^10^ Faculty of Education University of Cambridge Cambridge United Kingdom

## Abstract

The genetic counseling profession is continuing to develop globally, with countries in various stages of development. In some, the profession has been in existence for decades and is increasingly recognized as an important provider of allied health, while in others it is just beginning. In this article, we describe the current global landscape of the genetic counseling specialty field's professional development. Using examples of the United States, United Kingdom, Canada, Australia, South Africa, and various countries in Asia, we highlight the following: (a) status of genetic counseling training programs, (b) availability of credentialing through government and professional bodies (certification, registration, and licensure), and potential for international reciprocity, (c) scope of clinical practice, and (d) health‐care system disparities and cultural differences impacting on practice. The successful global implementation of precision medicine will require both an increased awareness of the importance of the profession of “genetic counselor” and flexibility in how genetic counselors are incorporated into each country's health‐care market. In turn, this will require more collaboration within and across nations, along with continuing engagement of existing genetic counseling professional societies.

## 1 INTRODUCTION

The profession of genetic counseling started in the United States with the advent of the first master's level training program at Sarah Lawrence College in New York in 1969. Since then, the profession has expanded globally, and in early 2018 we estimate there are nearly 7,000 genetic counselors in over 28 countries (see Table 1). In some countries (South America, many parts of Africa and Asia, and some European countries), physicians primarily provide genetic counseling; in some cases that is even a legal requirement as genetic counseling is considered a medical service. However, even in these countries, genetic service development is being forged with the input and support of genetic counselors who have often trained outside of their countries. Elsewhere around the world, the genetic counseling profession is in various stages of development; training programs are being established, and in some cases, forms of regulation and/or credentialing are being implemented, often leading to the national recognition of the profession.

**Table 1:** Global state of the genetic counseling profession

Region	Countries where genetic counseling exists as a profession^a^	2018 estimated number of GCs	Year of first established master's training program (total no. of programs)
North America	US, Canada	4400	1969 (42, with 5 more under review 1/2018)
Europe	Denmark, France, Ireland, Netherlands, Norway, Portugal, Romania, Spain, Sweden, Switzerland, UK	900	1992 (8)
Middle East	Israel, Saudi Arabia	<100	1997 (2)
Oceana	Australia, New Zealand	∼300	1995 (1‐year graduate diploma); 2008 (Masters) (2)
Africa	South Africa	∼25	1988 (2)
Asia	India, Indonesia, Japan, Malaysia, Philippines, Singapore, South Korea, Taiwan	350	2003 (5)
Central/South America	Cuba	∼900	1999 (1)

a Existence of the profession does not imply governmental acknowledgement of the profession or a regulatory process, but rather than the profession exists separate from physicians or other health‐care providers offering genetic counseling services. Other countries not listed have small numbers of genetic counselors trained in other countries who may be offering both clinical services or consulting services through corporate or academic laboratories.

In this article, we explore development of the genetic counseling profession using the four countries where genetic counseling is most well developed as a profession: United States, Canada, the United Kingdom, and Australasia (which includes Australia and New Zealand, but we will call Australia for the sake of simplicity). We will also highlight efforts in South Africa and across Asia. We discuss similarities and differences in training, scope of practice, and types of clinical services where genetic counselors practice, as well as country‐ and region‐specific issues (e.g., health‐care systems, culture) that shape the manner in which genetic services, including genetic counseling, are offered. We refer readers interested in a more granular summary of development of the genetic counseling across the globe to a paper by Abacan et al. (personal communication, February 5, 2018).

## 2 TRAINING

In the late 1980s, nearly 20 years after the profession of genetic counseling was established, approximately 15 genetic counseling masters programs existed in the United States. A certification examination had been developed in 1981 in conjunction with credentialing of medical geneticists, and work was underway to establish more rigorous and consistent criteria for genetic counseling training (Scott, Walker, Eunpu, & Djurdjinovic, 1988; Walker et al., 1990). This ultimately led to the establishment of the American Board of Genetic Counseling (ABGC) in 1993, which began certifying genetic counselors and accrediting training programs in the United States and Canada, a role now undertaken by the Accreditation Council for Genetic Counseling (ACGC) (https://www.abgc.net/about-abgc/detailed-history.aspx/, accessed January 25, 2018). During this period, the curriculum and clinical training requirements were established and continue in similar form even in 2018 as described by the current ACGC standards and practice‐based competencies (http://www.gceducation.org/Pages/Standards.aspx, accessed January 25, 2018).

Graduate‐level training programs in genetic counseling began in Canada in the mid‐1980s, and in Australia and the United Kingdom in the early 1990s. Canada (Leeming, 2013) and the United Kingdom (Skirton et al., 1998) adopted a master's degree from the start. In Australia, training started in 1995 as a 1‐year Graduate Diploma program and evolved in 2008 into the 2 year master's programs currently offered as the minimal entry requirement for certification (Barlow‐Stewart, Dunlop, Fleischer, Shalhoub, & Williams, 2015). South Africa started their first training program in 1988, based primarily on the U.S. training model for genetic counselors (Kromberg, Wessels, & Krause, 2013). Training programs in all these countries are similar in terms of general curricular requirements in scientific, clinical, and psychological areas, the incorporation of supervised clinical training and a research project with varying requirements and accreditation by professional governing bodies. As a result, there is international recognition between these countries of the master's degree qualification.

Asia is a diverse region, and the development of genetic counseling training has been equally diverse as the profession becomes established. China, India, Indonesia, Japan, Malaysia, Philippines, South Korea, and Taiwan currently offer graduate‐level genetic counseling training programs (Laurino et al., 2018), and some countries (e.g., India) have multiple approaches evolving in parallel. While some offer a 2 year master's degree in genetic counseling (e.g., India's Kasturba Medical College, Indonesia, Japan, Malaysia, Philippines, South Korea, Taiwan), others offer a 1‐year program or a 6‐month track as part of an already existing master's program (e.g., India's Vellore Institute of Technology offers a genetic counseling track as part of their master's degree in biomedical genetics). Understandably, these differences are due to varying available resources including access to experts to teach genetic counseling courses, and available budget for training program operational cost. Local leaders (medical geneticists, genetic counselors, and other allied health providers) actively advocating for genetic counselors needed to be creative in establishing training programs to meet their current demands. To illustrate, China's 1‐year program, launched in 2016, is offered as a joint collaboration with Peking University Health Science Center in Beijing, China and the University of Manchester, Manchester Centre for Genomic Medicine (MCGM, 2017). This Clinical Genetics and Genetic Counseling professional training course is provided through a combination of both e‐learning sessions and intensive 4‐day face‐to‐face case‐based genetic counseling training sessions in Beijing. Fostering partnerships amongst Asian countries, the Professional Society of Genetic Counselors in Asia (PSGCA) leadership recently convened with representatives from the Board of Genetic Counseling in India, the Indonesian Society of Genetic Counselors, the Japanese Board of Genetic Counseling, and the Taiwan Association of Genetic Counseling to begin work in aligning core skill requirements and curricular standards for genetic counselor training in the region.

The background and prior experience of students entering into the masters programs is another area of variation both between and within countries. Across all countries, students are expected to have a strong background in science and to demonstrate communication and empathy skills. While frequently this means students enter with their main degree in a scientific field, some countries will also allow well‐qualified students to enter with a psychology or social science degree if they meet other course prerequisites. Given that the university‐level training in some countries is very focused on the major area of study, it can be challenging for applicants from outside of the country to meet application prerequisites, and they may be required to take entrance examinations or document competence in basic principles in other ways. Within the United Kingdom, Australia, and South Africa in particular, it is preferable if students can demonstrate prior experience of working in a “caring role,” which means that many graduates from genetic counseling programs have also had previous life experiences or volunteer work in nursing, psychology, or other areas of medicine or social or crisis support services. The United States and Canada tend to focus on applicants who have had experience specifically providing one‐on‐one counseling (preferably in an organization that provides structured training around active listening skills), for example, crisis counseling or social support services. Some countries (e.g., Philippines, Indonesia, Taiwan, etc.) have also focused on individuals with prior clinical training as physicians, physician assistants, or nurses, as these professions may already be recognized as health‐care providers, allowing different clinical practice options. The opportunity to enroll in a genetic counseling training program provides these health‐care professionals the chance to increase their knowledge of the genomic contribution to health, and addresses gaps when there are no medical genetics training programs or limited training slots for practitioners who are not specifically trained in pediatrics.

## 3 CREDENTIALING, REGISTRATION, AND INTERNATIONAL “RECIPROCITY”

Table 2 summarizes the current state of credentialing and continuing education requirements in several countries. As our profession becomes increasingly global, and given workforce shortages of genetic counselors in some countries (e.g., Dobson and DaVanzo, 2016), the potential for genetic counselors to become credentialed through processes of certification, registration, or licensure in countries other than where they have trained has become a critical issue. These terms are often used interchangeably but they have slightly different meanings and may be implemented in different manners even when the credentialing intent is similar. Key to this discussion is understanding that credentialing can occur through a statutory (governmental) regulation, either as a state‐, province‐ or country‐based format, or through professional organizations.

**Table 2:** Global credentialing of genetic counselors

Region	Credentialing body	Eligibility criteria	Internationally trained option for credential?	Recertification?	State‐ or Province‐based registration or licensure
North America	ABGC (U.S. and some approved Canadian Programs)	Two year MS (Masters Genetic Counseling) from an ACGC accredited training program; 50 case logbook; pass certification exam to demonstrate entry level competency.	Yes, if attended an MS GC program that is considered accredited: http://www.abgc.net/becoming-certified/approved-accrediting-bodies/	Every 5 years by continuing education unit (CEU) or exam	Twenty states require licensure (typically based on ABGC certification); most employers require certification
	CAGC (Canada)	Two year MS from an ACGC accredited or Canadian training program; 50 case logbook; pass certification exam to demonstrate entry level competency	Yes, see https://www.cagc-accg.ca/?page=111	Every 10 years by CEU or exam	No, but under consideration
Europe	GCRB (UK)	2 year MS from a GCRB accredited training program plus 2 years experience as a genetic counselor OR 3 year combined MS and work‐based genetic counselor training; extensive portfolio to document competence (50 case logbook, evidence of counseling supervision; case studies and essays and video recorded consultations	Yes, see http://www.gcrb.org.uk/media/9332/overseas-guidelines-v2-jan-2017.pdf	Every 5 years by Continuing Professional Development reflective log	Voluntary but most employers only hire registered GC or those working toward. Statutory regulation is available for new 3 years combined MSc and work‐based genetic counselor training (“STP training”)
	EBMG:https://www.eshg.org/471.0.html	MS in GC, ideally from EBMG accredited program. Logbook of 50 cases, case studies, references, reflective essays, Grandfather clause	Yes, if registered in home country and after working fulltime in Europe for 1+ year	Every 5 years by CEU	Not required
Oceana	HGSA (Australia/NZ)	MS in GC from HGSA accredited program, 2 years of clinical practice, logbook of 50 cases and supervisor reports, 5 case studies, reflective essay, publication and simulated case.	Yes, varies by country: https://www.hgsa.org.au/documents/item/39	Voluntary by CEU	No but development of self‐regulation under the auspice of the HGSA in progress
Africa	HPCSA (South Africa)	MS in GC, 2 year internship (1 year completed after MS degree)	Yes, United Kingdom, Europe, and Australia are eligible; others on case‐by‐case basis.http://www.hpcsa.co.za/PBMedicalDental/Education	Yes, by CEU	Required
Asia	Japan Board of Genetic Counseling	MS in GC from an accredited program	Yes, as long as certified by ABGC, etc.; active member of JSGH and/or JSGC for at least 2 years For more info:http://tagc.med.sc.edu/documents/credentialing/JapanCredentialing.pdf; http://plaza.umin.ac.jp/~GC/Link.html	Yes, every 5 years (CEU)	Not required
	Taiwan Association of Genetic Counseling		http://www.taiwangc.org.tw/en/index.asp		
	Board of Genetic Counseling, India		http://www.geneticcounselingboardindia.com/index.html		

Only a handful of countries have a national statutory regulation of genetic counselors. The Health Professions Council of South Africa (HPCSA) governs the genetic counseling profession using Genetic Counselling South Africa (GCSA)‐developed standards of practice guidelines to guide the training and registration of genetic counselors in South Africa. Malaysia is currently the only Asian country wherein genetic counselor registration is in place with the Lembaga Kaunselor Malaysia (https://www.lkm.gov.my/).

In the United Kingdom, there is a state of flux, which is likely to be resolved throughout 2018; here, “registration,” for part of the profession at least, will also mean “regulation,” which entails a government supported licensure equivalent. Graduates from the new Masters Level genomic counseling degree apply for a Certificate of Attainment from the Academy for Healthcare Science (AHCS), which allows registration as a clinical scientist (genomic counselor) with the Health and Care Professions Council (HCPC). Registration with the HCPC comes with a legal protection for the title and government‐recognized competency to practice. The genetic counseling profession in the United Kingdom has been campaigning for statutory regulation for more than 10 years and at the moment, the mechanisms to transition from voluntary to statutory regulation (also called “registration”) are still being explored. The United Kingdom also has “registration” through a professional body, which is assessed via a portfolio of work submitted to the Genetic Counsellor Registration Board (GCRB) within the United Kingdom and Republic of Ireland (ROI).

In areas where the profession of genetic counseling is well‐established but it is not yet a legal requirement and no registration or licensure systems are in place or available, the professional societies and/or registration bodies have taken on this self‐regulatory role while advocating for appropriate legislation and/or statutory regulation. The terms “registration” and “certification” are both used to describe voluntary credentialing by professional bodies. For example, in the European Union (EU), the European Board of Medical Genetics (EBMG) provides genetic counselor registration through an EBGC portfolio process similar to that established by the GCRB in the United Kingdom. In United States, Canada, and Australia, the term “certification” is used to describe this professional body credentialing, but it is achieved through different means; in this case, via examination through ABGC or CAGC, or through portfolio application to the Human Genetics Society of Australasia (HGSA). Additionally, the United States also has been lobbying for statutory regulation and since the early 2000s has achieved state‐based licensure, a governmental regulatory process, in 20+ of the 50 U.S. states (https://www.nsgc.org/p/cm/ld/fid=19, accessed February 12, 2018). Pending federal recognition and regulation in the United States, state‐based licensure functions as a recognition process to ensure an individual is competent to practice in a specific profession (in this case as a genetic counselor), and is meant to ensure public safety and title protection for those within the profession. Requirements for licensure vary by state, but most have accepted the ABGC certification examination as documentation of competency. India, Taiwan, and Japan have also established a certification process with oversight by their in‐country professional genetic counseling societies, but many other countries have no form of credentialing yet established.

While a master's degree in genetic counseling is the minimum entry to the credentialing process for most countries where one exists, one of the most important conceptual differences is that some countries (United States, Canada) consider their credential to measure entry level competency, while most other countries with registration or certification processes require a minimum of 2 years of supervised clinical work practice, postmaster's degree, and consider their credential to measure the competence to work autonomously. The various systems place emphasis on different competencies and measure them differently; some countries (e.g., United Kingdom, EU, Australia, South Africa, etc.) measure work practice by an assessment of a portfolio of evidence, focusing on lived experience of genetic counseling in practice, demonstrating scientific knowledge, counseling skills, use of counseling supervision, and reflective practice. Notably, these portfolios require clinical practice, which impacts the ability of genetic counselors working in nonclinical settings to become credentialed. Other countries (e.g., United States, Canada, Taiwan, India, etc.) require the prospective candidate to pass an examination that demonstrates applied clinical knowledge including both scientific and counseling skills. Additionally, countries vary in whether they allow alternative degrees or training approaches to be considered for genetic counseling registration, particularly in areas where professionals obtained on‐the‐job training to practice as genetic counselors in the early stages of the profession. For example, in Europe, the professional bodies that support genetic counselors were keen to keep the two professional groups—genetic counselors and genetic nurses, together. As such, the credentialing bodies that offer voluntary registration for genetic counselors (e.g., the GCRB in the United Kingdom and ROI, and the EBMG/EBGC in the EU) therefore recognizes two different pathways to register as a genetic counselor—the first via completing a Master's Genetic Counselling program, the second via a nursing route, which includes Master's level genetic nursing in Europe, or senior nursing practitioner plus training in genetics and counseling in United Kingdom and ROI (Paneque et al., 2016; http://www.gcrb.org.uk/media/9339/applicant-guidelines-v3-july-2017.pdf).

In some countries, credentialing is a legal requirement in order to practice clinically as a genetic counselor, while it is not in others. However, the many differences between training and credentialing processes have made the recognition between countries of each other's credentials challenging, though with the global demand for a genetic counseling workforce it is an issue that must be addressed. International recognition began informally and on a case‐by‐case basis in the early 2000s, thus registered/certified genetic counselors wishing to relocate and work in a different country would contact the appropriate regulatory board and ask for their training and certification to be recognized. The Transnational Alliance of Genetic Counseling (TAGC) formed an International Committee on Genetic Counselor Credentialing in 2011 to examine and document some of these issues. The aim and scope of this group was merely to begin dialog in this space. Currently in most countries, the process of international recognition is rigorous, with the first formal criteria for true reciprocal registration established by the GCRB in 2006. This started with a recognition that genetic counselors registered with the relevant boards in Australia (HGSA), South Africa (HPCSA), and the United Kingdom (GCRB) would be able to receive individual registration (“certification”) in any of these countries without having to complete a whole new registration process. Several certification boards currently recognize Master's training programs overseas (see Table 2) with varying country‐based requirements, including a reduced portfolio in some cases, to fully meet requirements for examination or portfolio. The only true reciprocal arrangements between registration boards exist between United Kingdom, Australia, South Africa, and the EBMG (Genetic Nurse and Genetic Counsellor Branches).

## 4 SCOPE OF CLINICAL PRACTICE

In most countries, the scope of practice of master's‐trained genetic counselors includes working with patients (and their families) who face conditions with a genetic component in a clinical setting. The National Society of Genetic Counselors (NSGC) has framed the clinical scope of practice for genetic counselors to include medical roles (history taking; risk assessment; education regarding inheritance, natural history, and genetic testing; coordination of testing, including cascade testing, and in some cases ordering the genetic testing), psychosocial support (assessing adaptation, providing anticipatory guidance and short‐term client‐centered counseling) and case management (documentation; provision of resources) (https://www.nsgc.org/p/cm/ld/fid=18#scope, accessed January 22, 2018). These roles seem to occur at least in part across the globe by individuals trained as genetic counselors, though there is within and between country variation in how these roles are implemented, and specifically with regard to how psychotherapeutic the genetic counseling process is. Within the United Kingdom, Australia, and South Africa, genetic counselors aim to follow a patient‐centered psychotherapeutic process, integrating genetics/genomics knowledge into a consultation that is based on the clients' needs (Clarke et al., 2007; Middleton, Hall, & Patch, 2015). In the United States and Canada, though the importance of a “psychosocial” focus to the genetic counseling interaction has been recognized as a core, or foundational element (Veach, Bartels, & LeRoy, 2007; http://www.gceducation.org/Documents/ACGC%20Core%20Competencies%20Brochure_15_Web.pdf, accessed February 12, 2018), process studies show that genetic counselors tend to practice in a more didactic, teaching model‐based manner (Hartmann, Veach, MacFarlane, & LeRoy, 2015; Lerner et al., 2014; Meiser, Irle, Lobb, & Barlow‐Stewart, 2008; Roter, Ellington, Erby, Larson, & Dudley, 2006). Growing evidence suggests the best patient outcomes are associated with a more counseling‐based model (Redlinger‐Grosse et al., 2016), and this is currently pushing the profession in these countries toward refocusing on the psychotherapeutic aspects of the work (Austin, Semaka, & Hadjipavlou, 2014).

Specifically in Asia, an online survey conducted by the PSGCA was recently completed in order to gain a better understanding on the clinical scope of genetic counselors currently practicing in the region. In addition to being a valuable “genetics‐expert” resource to patients and families affected with genetic conditions, the genetic counselor's role in providing psychosocial counseling is highlighted as one of the main reasons of the profession's important contribution as a member of the health‐care team. Clearly distinguishing, as best as possible, the unique and value‐added role of genetic counselors in the region, and in fact globally, will help standardize the necessary core practice competencies for training and professional development.

Finally, genetic counselors across the globe work both as part of a health‐care team and in some cases in a more autonomous role, and they practice across various specialities that may or may not include medical geneticists. One area of variation is the frequency with which genetic counselors work as “generalists” as part of a medical genetics team (frequently seeing all types of genetics referrals), versus within a specialty team (e.g., in cancer genetics, cardiology genetics, etc.) that is increasingly embedded within that medical speciality and working in conjunction with nongenetics trained physicians who become experts in genetic conditions within their specialty. In the United States, specialty practice has been increasing rapidly over the past 20 years, with substantial percentages of genetic counselors reporting that they work in cancer genetics (48%), cardiogenetics (10%), neurogenetics (∼8%), infertility genetics (∼5%), and other speciality areas (NSGC Professional Status Survey, 2016). In Australia, many of the familial cancer services are led by medical oncologists who work alongside genetic counselors. More recently, however, with the decreasing costs of genetic testing, oncologists and surgeons working in private practice are ordering genetic tests. For some time this has been happening in the context of treatment focused genetic testing in breast and ovarian cancer (Quinn et al., 2017) with those women found to be mutation‐positive referred to familial cancer services. However it is anticipated that with genetic testing becoming recognized as an important component of medical care (“mainstreamed”), more nongenetics health‐care providers in other specialities will be ordering tests. Current limitations on genetic counselors charging a fee‐for‐service means that they are often not involved with these physicians clinically although they may be working in the laboratories providing testing and assisting with return of results. While the future role of genetic counselors in Australia as genetic testing is mainstreamed in this genomics era remains unclear, recent policy frameworks developed at the National and State levels recognize there is a clear need for their involvement (https://consultations.health.gov.au/genomics/national-health-genomics-policy-framework/supporting_documents/National%20Health%20Genomics%20Policy%20Framework%20Consultation%20Draft%20D161361443.PDF).

Generalist practice occurs more frequently in countries with smaller numbers of genetic counselors, because geography (e.g., rural clinics) and workforce limitations require them to address all referral indications. For example, in South Africa, genetic counseling is primarily available in the major cities and mostly occurs at tertiary health‐care service centers. Patients in rural areas have limited access to genetic counseling and services by telephone and videoconference have recently been implemented to address this. Only 2 of the 11 provinces of South Africa have genetic counselors, 2 more provinces have genetic services provided by specialists in medical genetics, and for the rest of the country, genetic services are provided by nongenetics health‐care providers (Greenberg, Kromberg, Loggenberg, & Wessels, 2012).

Another area of difference is the frequency with which genetic counselors work in clinical versus nonclinical roles across the various countries. In some countries, this has been a rapidly growing area for genetic counselor positions, with expansion into academic and commercial laboratory genetic counselor roles and research‐related genetic counselor roles, both clinical roles and nonclinical roles. The transition of genetic counselors into these nonclinical roles contributes to some degree to the workplace shortages mentioned above. In the United States, at least 20% of genetic counselors are employed in primarily industry‐based positions (NSGC Professional Status Survey, 2016), and in Canada ∼30% of genetic counselors are in nondirect patient facing roles (CAGC, 2016). Research, education and industry roles are emerging for genetic counselors in the United Kingdom (Middleton et al., 2017) and Australia (Barlow‐Stewart et al., 2015), and there is a slower rate of growth in South Africa and Asian countries, noting a few genetic counselors working primarily in industry (e.g., India, Hong Kong, Singapore, Thailand), but the majority practice clinically as “generalists” in both private and public health‐care settings across Asia.

## 5 HEALTH‐CARE SYSTEM DISPARITIES AND CULTURAL DIFFERENCES

An important global difference in how genetic counseling is practiced can be traced back to the different type of health systems that exist, including which types of patients can be seen autonomously, which services and tests are available and how they are offered, billed, ordered, and reimbursed. Accessibility to genetic testing is based on several issues: availability of testing (including laws that may govern whether testing may occur internationally or only in‐country), variation in what tests are covered by public and private payer systems, as well as who is permitted to order (request) tests. Below, we provide several examples of practice variation based on health‐care system, contrasting systems that are primarily publically funded (e.g., United Kingdom, Canada, Taiwan, South Korea) with those who have mixed systems (e.g., Australia, South Africa, Philippines, etc.) and those that are primarily private payer systems (United States). We will also discuss how these variations include the clinical incorporation of noninvasive prenatal screening (NIPS) and whole exome/genome sequencing (WES/WGS).

In the United States, health‐care services (both medical services such as genetic counseling and the genetic testing itself) are primarily funded through private payers, with around 25% of patients having publicly funded insurance. In recent years, genetic testing has moved from academic laboratories toward commercial laboratories, and this has accelerated the pace of clinical translation of new genomic technologies such as NIPS (first offered in 2011 to high‐risk women and more recently offered to all pregnant women), WES (2011) and next‐generation sequencing‐based gene testing panels (2013). The commercial drive for genetic testing in the United States means that different companies offer different testing options; for example, cancer panels across different companies include a wide range of genes with a range of evidence around clinical validity and clinical utility, and a wide range of pricing. Their broad conceptual availability does not mean that all patients are able to access these technologies, however, and many are faced with justifying genetic test orders with letters of medical necessity, with high deductible co‐payments and with denial of coverage.

In Canada, health‐care services are publically funded, with the government setting health‐care standards through the Canada Health Act, and through provincial funding for regional service delivery. Each province has its own health‐care insurance plan, and there is variation regarding which professions are regulated (sometimes with diverse legislative approaches) and their scope of practice. Similarly, the specific types of genetic testing (e.g., NIPT, WES) that are covered by the health‐care system, and for whom, may vary on a provincial/territorial basis. In the United Kingdom, most genetic counselors practice clinically within Regional Clinical Genetics Services (or the newly formed Genomic Medicine Centres) within the publicly funded National Health Service (i.e., genetic testing and genetic counseling is paid for by the government and not the patient). Genetic counselors work together with their clinical geneticist and clinical scientist colleagues as well as independently and autonomously with their own patient load. Although private practice for genetic counselors is on the increase, it is by no means the predominant role. As genomics becomes “mainstreamed,” that is, testing is offered throughout a whole health‐care setting, the roles for genetic counselors are evolving, with more involved in teaching, policy, research, and outreach clinical services (Middleton et al., 2017). With regard to genetic testing in both Canada and the United Kingdom, since the health service pays for genetic testing, decisions about which test to offer and to whom are based on medical necessity; each country has rigorous requirements for tests that are covered to be “medically necessary” or “required” (Canada Health Act) clinical utility and cost‐effectiveness before offering testing (https://sencanada.ca/content/sen/committee/372/soci/rep/repoct02vol6part7-e.htm, accessed February 12, 2018). In the United Kingdom, for example, NIPT is now available; whole genome sequencing (WGS) is only available within defined research projects, for example, in England via the 100,000 Genomes Project (Caulfield et al., 2017). However, the availability of other forms of genetic and genomic testing (e.g., a clinical exome, gene panel tests, virtual panel based on a WES or sequencing of a single gene) is based on the clinical question being asked.

Australia and South Africa are examples of a public/private mix of health‐care delivery, which raises concerns that future access to genetic testing will be increasingly available to those who have the capacity to pay, while waiting times for accessing publicly funded services will become longer. In Australia, genetics services are available in all States and Territories though the public health system. Where genetic testing is considered appropriate, it is offered free of charge, funded largely by the State Governments which are responsible for health‐care delivery. If a patient wishes to access genetic testing that is not available through the public system, it may be accessed privately. Genetic tests offered by physicians in private practice (a major component of the Australian health system) are largely on a fee‐for‐service basis. The private health insurance system does not cover the costs of the tests. Only a few tests are currently funded nationally with reimbursement to the patient through the national Medicare system, although that number is increasing. Similarly, in South Africa, some private medical insurance (self‐funded medical aid) schemes will cover local genetic testing (which is limited), others will cover a portion or none of the costs. In such cases patients are required to self‐pay for genetic testing. Because of the high costs of genetic tests in South Africa, tests are often performed by international laboratories that may cost up to 50% less than the local price. However, getting medical aids to cover the cost of international genetic testing is challenging. For example, access to array CGH only became locally available in 2017; gene panels and WES are available on a research basis in state laboratories or on a fee‐for‐service basis to individuals with private insurance, although the cost and funding remains problematic. Because there are a number of founder mutations for various genetic conditions in the local South African populations, founder testing is still being offered particularly through the state laboratory services. However, with the improvement in genetic testing technology it is becoming more cost‐effective to perform next‐generation sequencing on genes as opposed to testing for selective founder mutations.

While genetic testing companies offering NIPT/NIPS are actively marketing in the Asia region, the limited number of trained providers offering quality genetic counseling and overall low medical genetic literacy may compromise the informed decision‐making process (Chandrasekharan, Minear, Hung, & Allyse, 2014). The provision of genetic counseling services, in itself, differs among countries in Asia, and these underlying differences are attributed to country‐specific health‐care systems and honoring its cultural, religious, and ethical norms (e.g., pregnancy termination being legal in some countries and not in others) (Laurino, Sternen, Thompson, & Leppin, 2017). This being said, a trained genetic counselor is expected to maintain professional standards in providing the patient and their family genetics education and appropriate psychosocial counseling. But what may differ are the additional services/resources available to those individuals/patients. In general, the majority of genetic counselors in Asia provide clinical genetic counseling services or conduct research whereas a minority primarily work for genetic testing laboratories.

Finally, not surprisingly, cultural, linguistic, and religious issues also influence the provision of genetic counseling services. For example, South Africa has 11 official languages, while the Philippines has approximately 175 ethnolinguistic groups; this makes it challenging to provide counseling in a patient's native language. In addition, there are no words in many indigenous languages for “gene,” “chromosomes,” and “genetics,” making it difficult to follow the traditional western genetic counseling model. Research has also shown that across the world individuals have different worldviews and cultural practices, and these may differ significantly from a westernized and more biomedically focused approach to practice (Abad et al., 2014; Penn & Watermeyer, 2012; Penn, Watermeyer, MacDonald, & Moabelo, 2010). And finally, religion and culture may also significantly impact the availability of various relevant services, such as the legal availability of pregnancy termination worldwide, impacting prenatal genetic counseling practice. Therefore, a critical aspect of genetic counseling is that the communication and services should be adapted to meet the needs of the individual and family, fostering of cultural competence in an attempt to address potential barriers (Saleh & Barlow‐Stewart, 2005; Yeo et al., 2005).

## 6 CONCLUSIONS

Genetic counseling is a rapidly growing profession with the overarching goal to add value to the care of patients with genetic conditions and their families. There are many global similarities in the educational process, mechanisms of credentialing, and the scope of practice, but the profession has evolved in unique ways in different countries due to varying health‐care systems, legal restrictions, and cultural issues. The era of precision medicine is further challenging the way that genetic testing is offered, and the roles that genetic counselors play; thus far a “one size fits all” definition of the job title “genetic counselor” does not exist. Genetic counselors can learn from each other, sharing experiences, building on what works in other countries and adapting it to unique circumstances in one's own home country in order to improve care for our patients and their families. Together, we can be solution‐driven in strategically increasing professional recognition—both within and across nations.

## CONFLICT OF INTEREST

None.
